# Effects of CD4 Binding on Conformational Dynamics, Molecular Motions, and Thermodynamics of HIV-1 gp120

**DOI:** 10.3390/ijms20020260

**Published:** 2019-01-10

**Authors:** Yi Li, Lei Deng, Li-Quan Yang, Peng Sang, Shu-Qun Liu

**Affiliations:** 1 State Key Laboratory for Conservation and Utilization of Bio-Resources in Yunnan & School of Life Sciences, Yunnan University, Kunming 650091, China; yili@dali.edu.cn (Y.L.); uitb_dl@163.com (L.D.); 2 College of Mathematics and Computer, Dali University, Dali 671003, China; 3 College of Agriculture and Biological Science, Dali University, Dali 671003, China; ylqbioinfo@gmail.com

**Keywords:** molecular dynamics simulation, conformational transition, free energy landscape, conformational selection, structure-dynamics-function relationship

## Abstract

Human immunodeficiency virus type-1 (HIV-1) infection is triggered by its envelope (Env) glycoprotein gp120 binding to the host-cell receptor CD4. Although structures of Env/gp120 in the liganded state are known, detailed information about dynamics of the liganded gp120 has remained elusive. Two structural models, the CD4-free gp120 and the gp120-CD4 complex, were subjected to µs-scale multiple-replica molecular dynamics (MD) simulations to probe the effects of CD4 binding on the conformational dynamics, molecular motions, and thermodynamics of gp120. Comparative analyses of MD trajectories in terms of structural deviation and conformational flexibility reveal that CD4 binding effectively suppresses the overall conformational fluctuations of gp120. Despite the largest fluctuation amplitude of the V1/V2 region in both forms of gp120, the presence of CD4 prevents it from approaching the gp120 core. Comparison of the constructed free energy landscapes (FELs) shows that CD4 binding reduces the conformational entropy and conformational diversity while enhancing the stability of gp120. Further comparison of the representative structures extracted from free energy basins/minima of FELs reveals that CD4 binding weakens the reorientation ability of V1/V2 and hence hinders gp120 from transitioning out of the liganded state to the unliganded state. Therefore, locking gp120 conformation via restraining V1/V2 reorientation with small molecules seems to be a promising strategy to control HIV-1 infection. Our computer simulation results support the conformational selection mechanism for CD4 binding to gp120 and facilitate the understanding of HIV-1 immune evasion mechanisms.

## 1. Introduction

The viral envelope spike (Env), which is the infection machine of HIV type I (HIV-1), is a trimeric assembly composed of three external glycoprotein gp120 subunits and three transmembrane glycoprotein gp41 subunits [1]. The virally encoded glycoprotein gp120 is located on the viron’s surface and is therefore not only responsible for interactions with the host receptors, but is also the sole target of neutralizing antibodies. In contrast to a general membrane fusion mechanism of enveloped viruses, HIV-1 evolves a special two-step infection strategy to protect its conserved functional sites from attack by antibodies via successively binding to the receptor CD4 and the coreceptor CCR5/CXCR4 on the host cell’s surface [2,3]. Binding of CD4 by gp120 has been shown to play an intermediate role in viral infection through inducing large gp120 structural arrangements that promote the maturation of the CD4 binding site (CD4bs) and exposure of the coreceptor binding site [4]. Moreover, CD4bs-induced antibodies can directly interrupt interactions between gp120 and CD4 and hence prevent virus entry [5]. Interestingly, in the newly developed clinical trial it has been shown that the combination of various anti-HIV-1 antibodies can maintain long-term viral suppression [6].

Accumulating structural [7,8,9,10,11,12] and biophysical [13,14] studies indicate that the CD4-triggered conformational arrangements in gp120 are necessary for coreceptor recognition and subsequent virus-cell membrane fusion. In 2015, Rasheed et al. generated a full-length structural model of gp120 in complex with CD4 and antibody 17b using a computational protocol that integrates available X-ray structures, cryo-electron microscopy (cryo-EM) maps, and information of binding interactions [10]. A recently reported cryo-EM structure of the CD4-bound gp120 [12] reveals a dramatic displacement of the V1/V2 region when going from the unliganded state to the liganded conformation. Evidence for large conformational changes in gp120 upon CD4 binding also comes from the energetics experiments [13], which reveal an unexpectedly large magnitude of binding thermodynamics, including changes in enthalpy, entropy, and heat capacity upon CD4 binding. The hydrogen-deuterium exchange (HDX) technique, which measures the rates of deuterium incorporation into backbone amides under solution condition, has been employed to characterize CD4-induced conformational changes in a soluble HIV-1 Env trimer [14], revealing that CD4 binding induces reorganizations of the V1/V2 region, V3 loop, and much of the inner domain of gp120. However, HDX cannot provide detailed information on the conformational flexibility of a protein because it is only based on the hypothesis that the regions undergoing rapid deuterium exchange are unstructured or flexible, while those involved in stable hydrogen-bonding networks (regular secondary structure regions) or those that are highly occluded from the solvent are rigid.

Interestingly, single-molecule fluorescence resonance energy transfer (smFRET) has revealed that the unliganded gp120 in the context of native Env trimers on the viral surface is intrinsically dynamic and capable of sampling at least three distinct conformations, i.e., the low-, intermediate-, and high-FRET states, without induction by any receptor or coreceptor [15]; further comparison of the probability distributions of the conformational states sampled by the unliganded Env (in the absence of a receptor/coreceptor) and the liganded Env (in the presence of CD4 and 17b) suggests that the low- and intermediate-FRET states are the ground (or unliganded) state and the CD4/17b-liganded state, respectively, for which the structures have been determined by cryo-EM [16,17,18]. The high-FRET state stabilized by CD4 is an intermediate during the conformational transition between the ground and CD4/17b-liganded states, but its structure has not yet been characterized. The smFRET results imply that CD4 could selectively bind to the intermediate-FRET state, thus disrupting the distribution of three states and shifting the equilibrium toward the liganded state. However, detailed questions on dynamic aspects of gp120-CD4 interactions, such as how CD4 stabilizes the liganded state and what the effects of CD4 binding would be on the conformational dynamics, molecular motions, and thermodynamics of gp120 remain unanswered.

Molecular dynamics (MD) simulations can provide an atom-level picture of protein dynamics and a representation of the free energy landscape (FEL) near the native state of a protein. Thanks to graphics processing unit (GPU)-accelerated computing, multiple-replica MD simulations [19] can now gain adequate sampling convergence. In this work, two simulation systems, one starts with the monomeric gp120 in the liganded state (hereafter referred to as the CD4-free gp120) and the other with gp120 in complex with the CD4 D1 (CD4_D1_) domain (hereafter, the gp120 in the complex is referred to as the “CD4-complexed gp120”), were constructed based on the recently published full-length gp120 model in complex with CD4 and 17b [10]. Aiming to probe the effects of CD4 binding on the conformational dynamics, molecular motions, and thermodynamics of gp120, we performed a series of multiple-replica MD simulations on both systems, with the total simulation period reaching 2 µs. Our results reveal that CD4 binding suppresses the conformational fluctuations of gp120, while CD4 removal allows gp120 to transition to the unliganded state, thus providing a basis by which to better understand the mechanisms of receptor association and HIV-1 immune evasion.

## 2. Results

### 2.1. Molecular Architecture of gp120

**Figure 1:**
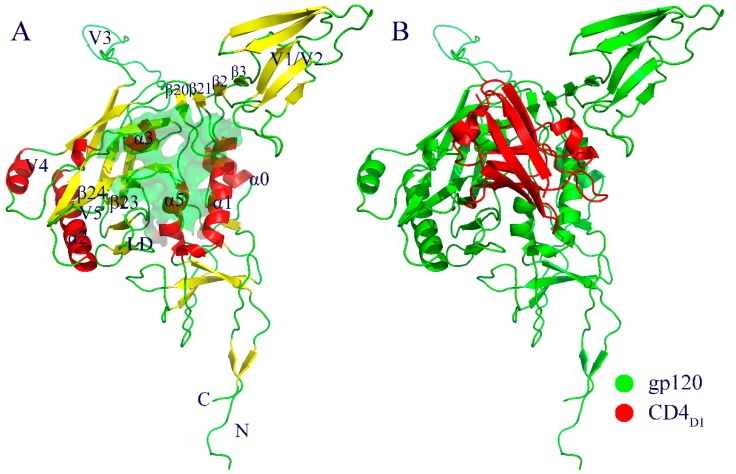
Cartoon representations of the structural models used as starting points for molecular dynamics (MD) simulations. (**A**) CD4-free gp120. (**B**) gp120-CD4 complex. In (**A**), α-helices, β-strands, and loops are colored red, yellow, and green, respectively, and the gp120-CD4 interaction interface is represented as a semi-transparent green surface. In (**B**), gp120 and the CD4 D1 domain (CD4_D1_) are colored green and red, respectively.

Structural models of the CD4-free gp120 and gp120-CD4 complex were extracted from the HIV-1 Env model (PDB ID: 3J70 [10]). Figure 1 shows the cartoon representations of these models. It should be noted that gp120 in both models is full length and in the liganded state. The molecular architecture of the liganded gp120 can be divided into five surface-exposed variable regions (V1-V5), a bridging sheet, and a conserved core. The structural core can further be divided into two domains, the inner domain and the outer domain, with the inner domain mainly composed of a near-terminal seven-stranded β-sandwich and three α-helices (α0, α1, and α5), and the outer domain of two end-to-end stacked β-barrels and two α-helices (α2 and α3). It is clear from Figure 1 that both the V1/V2 region and V3 loop protrude away from the gp120 core and present a Y-like orientation with respect to each other. The bridging sheet is composed of four anti-parallel β-strands in the order β3-β2-β21-β20, in which the β20–β21 hairpin has been considered as a regulatory switch for conformational transitions of HIV-1 Env [20]. According to the crystallographic structure of gp120 in complex with CD4 and 17b (PDB ID: 1GC1) [7], residues involved in direct contact with CD4 are distributed over four non-continuous segments of the sequence, including the CD4-binding loop (α3), a part of the ℒD loop, the tip of the β20–β21 hairpin in the bridging sheet, and parts of β23 and β24 in the outer domain.

### 2.2. Conformational Dynamics

**Figure 2:**
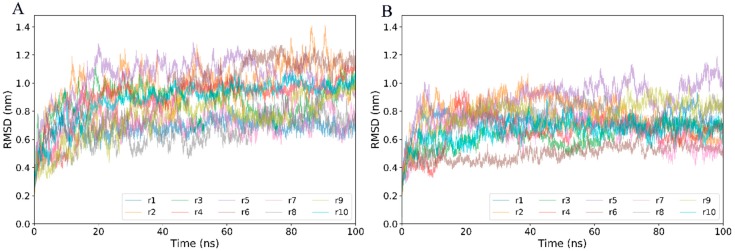
Time evolution of backbone root-mean-square deviation (RMSD) values, with respect to the starting structure calculated from 10 replicas (r1-10). (**A**) CD4-free gp120. (**B**) CD4-complexed gp120.

To evaluate the stability and equilibration of MD simulations, the time evolution of backbone root-mean-square deviation (RMSD) with respect to the starting structure was calculated (Figure 2). For both systems, all replicas show a rapid increase in RMSD values from the start of simulations until reaching a plateau after about 20 ns. For the CD4-free and CD4-complexed gp120s, the equilibrium portions of RMSD curves range between 0.5 and 1.2 nm and between 0.4 and 1.0 nm, respectively. When compared to the CD4-free gp120, the narrower range of RMSD curves for the CD4-complexed gp120, together with its smaller amplitude of RMSD fluctuations, indicates that CD4 binding suppresses the global structural deviation/change of gp120.

It has been shown that multiple-replica MD simulations can enhance conformational sampling of a protein by sampling different directions in the conformational space from the starting structure [19,21]. For each simulation system, only the equilibrated portions (20–100 ns) of the 10 replicas were concatenated into a single joined 800 ns trajectory, based on which the following analyses were performed to ensure that the calculated parameters reflected the intrinsic properties of gp120.

### 2.3. Conformational Flexibility

**Figure 3:**
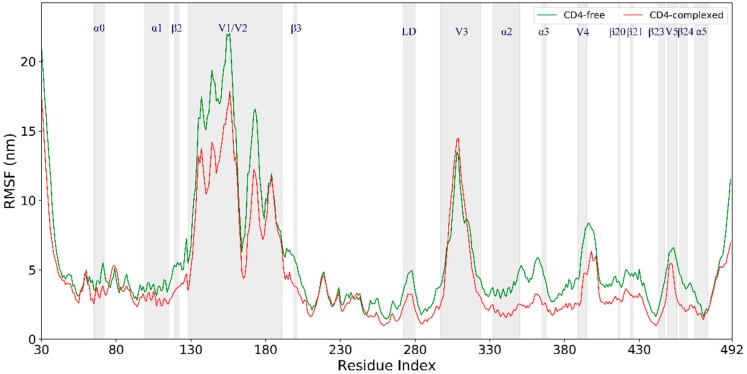
Root-mean-square fluctuation (RMSF) values of the C_α_ atom as a function of the residue number for the CD4-free (green line) and CD4-complexed (red line) gp120s.

Per-residue C_α_ atom root-mean-square fluctuation (RMSF) values (Figure 3) were calculated based on the joined equilibrium trajectories to evaluate and compare the conformational flexibility of these two gp120 forms. The variations in RMSF values along the chain of the CD4-free and CD4-complexed gp120s are very similar, with regular secondary structure regions exhibiting low values while the N-, C-termini, and variable loops show high values. Nevertheless, with few exceptions, almost all structural regions have higher RMSF values in the CD4-free gp120 than in the CD4-complexed gp120, resulting in the average RMSF values of 0.8 ± 0.20 and 0.6 ± 0.15 nm, respectively. Therefore, the CD4-complexed gp120 is characterized overall by lower flexibility/mobility as compared to the CD4-free form in MD simulations.

Close inspection of Figure 3 reveals that the regions participating in the formation of CD4bs, including the ℒD loop, the segment ranging from α2 to α3, β20–β21 hairpin, and the segment from β24 to α5, exhibit significantly lower RMSF values in the CD4-complexed gp120 than in the CD4-free gp120. This is not surprising, as the presence of CD4 restricts the free fluctuations of these regions. Surprisingly, some of the surface-exposed regions, such as layer 1 (mainly involved in α0), α1, V1/V2 stem (residues 120–128 and 194–203), and loops V4 and V5, also have somewhat lower RMSF values in the CD4-complexed gp120. The lower mobility of these regions in the CD4-complexed gp120 could also be attributed to CD4 binding, because they are located in the vicinity of CD4_D1_, whose large size may obstruct fluctuations of the nearby regions in gp120. Also worth noting is the V1/V2 region; although it extends away from the gp120 core and is distant from CD4_D1_, higher mobility was observed in the CD-free gp120. However, the other side of the Y-like structure, i.e., the V3 loop, exhibits very similar conformational flexibility in both gp120 forms. In summary, the binding of CD4 not only heavily restrains the conformational flexibility of CD4bs, but also imposes the inhibition effect on the flexibility of its nearby regions and even those relatively remote from CD4_D1_, ultimately resulting in a decrease in the global conformational flexibility of the CD4-complexed gp120 when compared to the CD4-free form.

### 2.4. Collective Motions

**Figure 4:**
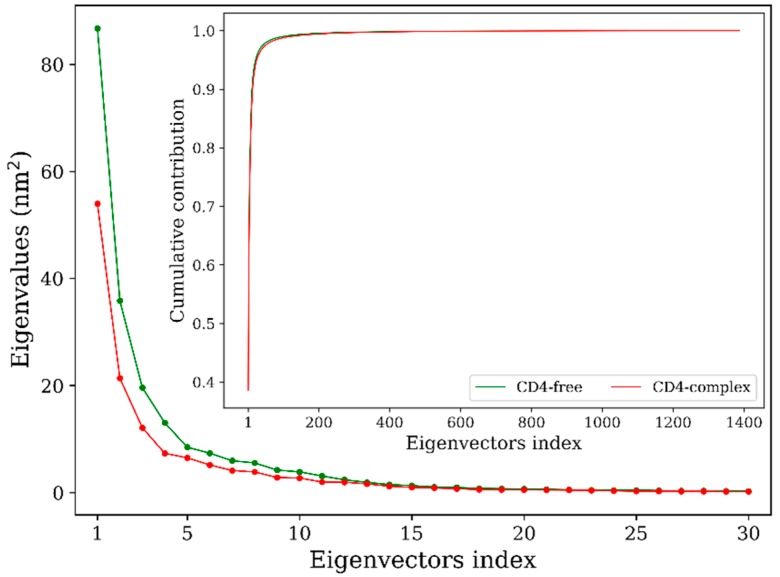
Eigenvalues as a function of eigenvector index obtained from principal component analyses of the joined equilibrium trajectories of the CD4-free (green line) and CD4-complexed (red line) gp120s. Main plot shows the eigenvalues of only the first 30 eigenvectors; inset plot is the cumulative contribution of all eigenvectors to the total mean square fluctuations.

Principal component analyses (PCA) of the joined equilibrium trajectories indicate that only a few eigenvectors possess eigenvalues which are large enough (Figure 4, main plot). Diagonalization of the covariance matrices gives the total mean square fluctuation (MSF) values of 216.8 and 139.8 nm^2^ for the CD4-free and CD4-complexed gp120s, respectively, indicating that the former experienced larger-amplitude fluctuations than the latter during simulations. Moreover, for the CD4-free gp120, the first three and five eigenvectors contribute 65.6% and 75.5% to the total MSF, respectively, and for the CD4-complexed form, the corresponding contributions are 62.6% and 72.5%, respectively. Therefore, collective atomic fluctuations along the first three eigenvectors represent the largest-amplitude protein motions.

**Figure 5:**
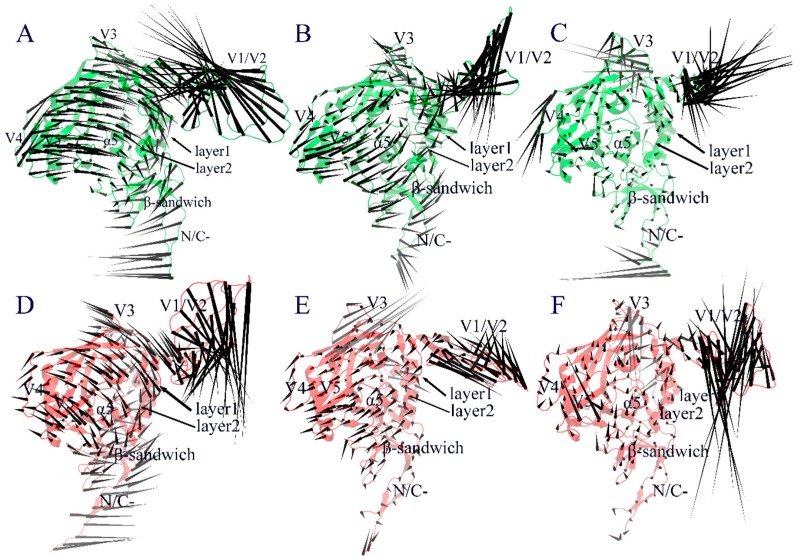
Porcupine plots showing the collective motions along the first three eigenvectors of gp120 in the CD4-free (**A**–**C**) and CD4-complexed (**D**–**F**) forms.

Figure 5 shows the collective motions of both forms of gp120 along the first three eigenvectors using the porcupine plot, in which the direction and length of the cone drawn on a C_α_ atom represent the fluctuation direction and amplitude of this atom along the eigenvector, respectively. As shown in Figure 5A, the first eigenvector of the CD4-free gp120 describes a common rotation of the inner and outer domains (which constitute the gp120 core) in an anticlockwise direction around an axis parallel to both domains; the V3 loop and N-, C-termini rotate in the same direction as that of the core, while the V1/V2 excursion moves with the largest amplitude in the up-left direction, leading to an approach to the distal end of the gp120 core. In contrast, the first eigenvector of the CD4-complexed gp120 (Figure 5D) describes a clockwise rotation of the gp120 core, and at the same time, V1/V2 moves in the down-right direction, exhibiting a trend of slightly more departure from the core. Along the second eigenvector, the core of the CD4-free gp120 (Figure 5B) exhibits somewhat similar anticlockwise rotation to that along the first eigenvector, but the V1/V2 region moves in the down-left direction, leading to its approach to the proximal end of the gp120 core. For the CD4-complexed gp120 (Figure 5E), it is obvious that the collective fluctuations of its structural core is somewhat restrained by CD4 binding, while V3 and V1/V2 exhibit the largest fluctuation amplitude, with the former moving in the opposite direction relative to that of the core and the latter displaying a self-contraction behavior. Along the third eigenvector, the core of both gp120 forms exhibits reduced fluctuation amplitude compared to that along the first two eigenvectors, except for some surface-exposed loop regions such as loops V3 and V4 in the CD4-free gp120 (Figure 5C) and V3 and V5 in the CD4-complexed gp120 (Figure 5F). Nevertheless, the reduced collective motions seem to lead to a slight expansion and contraction of the CD4-binding cavity in the CD4-free and CD4-complexed gp120 forms, respectively. Of note is that the V1/V2 excursion still has the largest fluctuation amplitude, but manifests as a twisting motion in the CD-free gp120 and up-and-down mixed motions in the CD4-complexed gp120.

### 2.5. Free Energy Landscape (FEL) and Representative Structures

**Figure 6:**
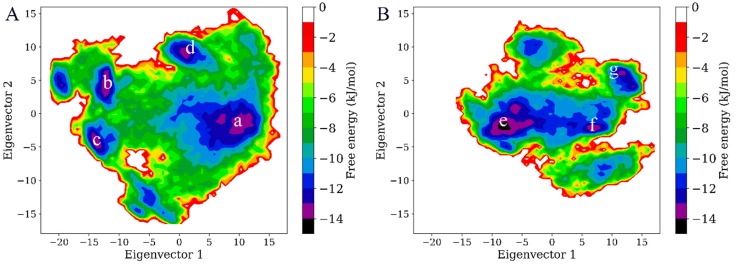
Constructed free energy landscapes (FELs) of the two forms of gp120 using projections of eigenvectors 1 and 2 as the reaction coordinates. (**A**) FEL of the CD4-free gp120. (**B**) FEL of the CD4-complexed gp120. The color bar indicates the relative free energy value in kJ/mol. The basins/minima with free energy level lower than −13 kJ/mol are labelled “a” to “d” in (**A**) and “e” to “g” in (**B**).

To reveal the effect of CD4 binding on the thermodynamics of gp120, the FELs for both forms of gp120 were constructed using the probability density function with the projection of joined equilibrium trajectories onto the first two eigenvectors as the reaction coordinates. As shown in Figure 6, both FELs present an irregular and divergent shape, distinguishing from the classical funnel-like FEL of the globular protein. In the FEL of the CD4-free gp120, there are four free energy basins/minima with an energy level lower than −13 kJ/mol (labelled as “a” to “d” in Figure 6A), while only three basins which have an energy level lower than -13 kJ/mol can be found in the FEL of the CD4-complexed gp120 (labelled as “e” to “g” in Figure 6B). If those with a free energy level lower than −10 kJ/mol are included, there are still more basins in the FEL of the CD4-free gp120 than of the CD4-complexed gp120, indicating there are more conformational substates sampled by the CD4-free gp120. In addition, the FEL of the CD4-free gp120 covers a larger region in the essential subspace than that of the CD4-complexed gp120, implying a large conformational entropy of the CD4-free gp120. Also worth noting is that the minimum free energy value of the CD4-complexed gp120 FEL is −14 kJ/mol (basin “e” in Figure 6B), which is lower than that of the CD4-free gp120 FEL, −13 kJ/mol, and this implies a higher stability of the CD4-complexed gp120. Taken together, the FEL of the CD4-complexed gp120 is characterized by fewer local free energy minima, larger free energy surface, and lower global minimum free energy value when compared to that of the CD-free gp120, indicating that CD4 binding reduces the conformational diversity and entropy and enhances the stability of gp120. 

**Figure 7:**
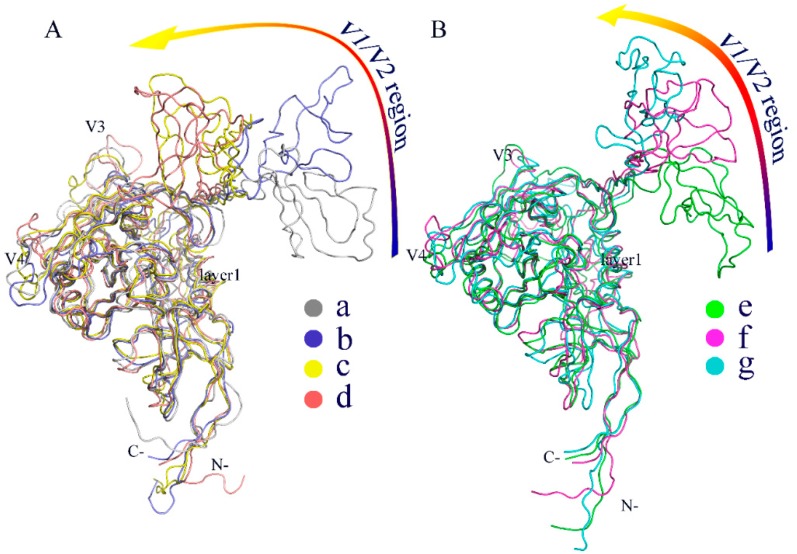
Backbone superimposition of the representative structures/substates extracted from the free energy basins/minima in the constructed FELs for (**A**) the CD4-free gp120 (“a” to “d”) and (**B**) the CD4-complexed gp120 (“e” to “g”).

To further compare the differences in conformation between thermodynamically different substates, the representative structures/substates of the two gp120 FELs were extracted from the low free energy basins as labelled in Figure 6. Figure 7A,B show the superimposed backbones of the four and three representative structures for the CD4-free and CD4-complexed gp120s, respectively. It is clear that for both gp120 forms, the cores of their representative structures show relatively small conformational differences, although those of the CD4-free gp120 are characterized by somewhat larger variations than the CD4-complexed form, especially in the core peripheral regions, such as V3, V4, layer 1, and N-, C-termini. The most pronounced differences were observed in the orientation of the V1/V2 region with respect to the gp120 core. The orientation of V1/V2 is similar between the starting conformations of both gp120 forms, as shown by the substates “b” and “f”, in which the V1/V2 tip points away from the gp120 core. However, in the substates “c” and “d” of the CD4-free gp120 (Figure 7A), the V1/V2 region is located in the vicinity of the V3 loop and partially covers the bridging sheet, indicating that the CD4-free gp120 sampled the conformations similar to the unliganded state during the multiple-replica MD simulations. In the case of the CD4-complexed gp120, although the orientations of V1/V2 are distinctly different among the three substates (“e” to “g”), V1/V2 still remains distant from the core, suggesting that the presence of CD4 prevents gp120 from transitioning toward the unliganded state. As a result, it can be concluded that: (i) the major differences among the sampled substates arise mainly from the distinct orientations of the V1/V2 region with respect to the gp120 core; (ii) CD4 binding locks gp120 conformation in the liganded state; and (iii) the removal of CD4 allows gp120 to transition between the liganded and unliganded states, in agreement with the results of the smFRET study [15].

## 3. Discussion

Previous X-ray crystallographic studies [7,22,23] have shown that the gp120 core assumes distinctly different conformations before and after CD4 binding. These crystal structures, together with the data from the energetics [13] and HDX [14] experiments evidencing the drastic conformational rearrangements of gp120 upon CD4 binding, support the induced-fit viewpoint that it is the CD4 binding that induces the conformational transition of gp120 from the unliganded to the liganded states. However, direct observations of conformational dynamics of gp120 in the absence of CD4 using smFRET [15] identified three distinct conformational states, which coexisted in equilibrium but whose relative populations were remodeled by CD4 and antibody binding. Such observations support the conformational selection mechanism [24] of CD4 binding, since the liganded state of gp120 has already existed, although with a low probability, in the conformational ensemble of the ligand-free gp120, and CD4 can bind selectively to this state and ultimately shift the equilibrium toward the liganded state. In the current study, we have performed multiple long-time MD simulations on gp120 in the liganded state with and without CD4 to probe the effect of CD4 binding on the dynamics and thermodynamics of gp120. Our results reveal that although the presence of CD4 suppresses the overall conformational fluctuations/flexibility of gp120 and hence locks gp120 in the liganded state, the removal of CD4 enhances the flexibility of gp120, allowing it to sample a wider conformational space and to transition backward to the unliganded state. The capability of the CD4-free gp120 in the initial liganded state to sample the other conformations including the unliganded state, together with the experimentally observed multiple states for the ligand-free HIV-1 Env trimer/gp120, indicates that conformational selection does indeed dominate the CD4 binding process.

Our MD simulations also identify the high-flexibility regions, collective motion modes, and the crucial regions directly involved in conformational transition of gp120, which could facilitate the understanding of the viral entry and immune evasion mechanisms of HIV-1. The observed high-flexibility regions are common to both forms of gp120, i.e., the V1/V2 region, loops V3, V4, and V5, and N-, C-termini (Figure 3). It has been shown that the high flexibility/mobility of the protruding V3 in the liganded state is advantageous for gp120 to sense and trap coreceptor molecules such as CCR5/CXCR4 [25,26], whose engagement will promote additional conformational changes to trigger the formation of gp41-entry machinery [3]. Because V4 is connected to the bridging-sheet element β20 through β19, and V5 is located between β23 and β24, the higher flexibility of these two loops in the CD4-free gp120 may explain the increased mobility of the bridging sheet as well as the β-strands connected by them, as compared to the CD4-complexed gp120. It is possible that the unstable bridging-sheet element would facilitate the disintegration of the mature bridging sheet followed by element rearrangements to obtain the premature one observed in the unliganded gp120 [27]. On the other hand, the highly mobile bridging sheet and outer-domain β-strands in the CD4-free gp120 likely impart increased lability to certain antibody neutralization epitopes (i.e., CD4-induced and CD4-binding-site epitopes), which may disturb recognition/binding of relevant antibodies to gp120 in the liganded state but without CD4 occupation.

For both forms of gp120, the V1/V2 region exhibits the highest flexibility during MD simulations (Figure 3). This is likely due to its complete departure from the core in the liganded state (Figure 1). Moreover, the V1/V2 region exhibits the largest fluctuation amplitude and complicated motion modes along the first three eigenvectors (Figure 5). However, no matter what the mode is and regardless of its complexity, the V1/V2 region of the CD4-free form shows a trend of moving toward the gp120 core, while in the CD4-complexed gp120, V1/V2 moves either upward or downward along the core side without a trend of approaching the core. In fact, of the four populated substates sampled by the CD4-free gp120, the two substates (“c” and “d” in Figure 7A) show the orientation of V1/V2 in proximity of V3 and partially covering the bridging sheet. In all three populated substates sampled by the CD4-complexed gp120, the V1/V2 region still stays distant from V3 and the bridging sheet, although its orientation, relative to the core, is distinct among the three substates. As a result, it is reasonable to consider that: i) V1/V2 is a key determinant in distinguishing among different conformational substates of gp120; ii) the reorientation ability of V1/V2 is crucial for gp120 to transition from the liganded to unliganded states; iii) CD4 binding weakens the reorientation ability of V1/V2 and hence prevents the restoring transition of gp120 to the unliganded state. Additionally, since the V1/V2 region participates in associations among the three gp120 subunits in the context of the unliganded Env trimer, its dynamical properties can affect Env stability and gp120 conformational transition (i.e., from the unliganded to the liganded states), and hence contribute to regulating the neutralization phenotypes of primary HIV-1 isolates [28,29,30].

## 4. Materials and Methods

### 4.1. Simulation System Preparation

Atomic coordinates of the CD4-free gp120 and gp120-CD4_D1_ complex were extracted from the structural model of HIV-1 Env (PDB ID: 3J70 [10]), in which the full-length gp120 subunits are in complex with CD4 and 17b. The reason for retaining only the CD4 D1 domain in the complex model is that among the four domains (D1 to D4) of CD4, only the D1 domain makes direct interatomic contact with gp120 [7] and, furthermore, its presence is necessary and sufficient for the induction of virus-cell membrane fusion [31,32]. First, the two structures were subjected to energy minimization in a vacuum to refine the models and to relieve strain resulting from any bad contact. Subsequently, these two energy-optimized structures were individually solvated in a dodecahedron periodic box of TIP3P water molecules [33], with a minimum solute-box edge distance set to 8 Å and numbers of Cl^−^ and Na^+^ introduced to obtain the electroneutral system at 150 mM salt concentration. Finally, each solvated system was once again subjected to energy minimization until no significant energy changes could be detected.

### 4.2. MD Simulations

All MD simulations were performed using GROningen MAchine for Chemical Simulations (GROMACS) 5.1.4 [34] with the AMBER99SB-ILDN force field [35]. Before production runs, each prepared protein-solvent system was subjected to four successive 200 ps position-restrained MD simulations with protein-heavy atoms restrained by decreasing harmonic force constants (i.e., 1000, 100, 10, and 0 kJ/mol/nm^2^) to soak the solute into water molecules [36]. To improve conformational sampling, ten independent 100 ns production runs were performed for each system, with each run initialized with different initial atomic velocities assigned from a Maxwell distribution at 300 K. In the production MD simulations, the following protocols were used: The LINear Constraint Solver (LINCS) algorithm [37] was used to restrain bond lengths to their equilibrium positions; the integration time step was 2 fs; system coordinates were saved every 2 ps; the particle-mesh Ewald (PME) algorithm [38] was used to treat long-range electrostatic interactions; a twin-range cut-off (1.0 and 1.4 nm) was used to treat van der Waals interactions; temperatures of the solute and solvent were separately coupled to a 300 K heat bath with a coupling constant $\tau_{t}$ of 0.1 ps; and pressure was maintained at 1 atm using the Parrinello-Rahman barostat [39] with a coupling constant $\tau_{p}$ of 0.5 ps.

### 4.3. Analysis Methods

RMSD and RMSF were calculated using GROMACS tools “gmx rmsd” and “gmx rmsf”, respectively. PCA was performed on the covariance matrix built from C_α_ atomic fluctuations in a MD trajectory, obtaining a set of eigenvectors and eigenvalues. Collective motion modes along the first few eigenvectors were shown as porcupine plots, which were obtained using a modevectors.py script with the two extremes of an eigenvector projection as the input. FEL with eigenvectors 1 and 2 as the reaction coordinates was constructed using the probability density function $F\left(s\right)=-k_{B}T\ln\left(N_{i}/N_{max}\right)$, where $k_{B}$ is the Boltzmann’s constant, T is the temperature, $N_{i}$ is the population of bin i, and $N_{max}$ is the population of the most populated bin.

## 5. Conclusions

In this work, we performed μs-scale multiple-replica MD simulations on the CD4-free gp120 and gp120-CD4 complex to probe the effects of CD4 binding on the conformational dynamics, molecular motions, and thermodynamics of gp120. Comparative analyses of the joined equilibrium trajectories in terms of RMSD and RMSF reveal that CD4 binding suppresses the global structural deviation/change, as well as the overall conformational flexibility of gp120. Further comparison of the constructed FELs indicates that CD4 binding reduces the conformational entropy and conformational diversity while enhancing the stability of gp120. The collective motions of the structural core in both forms of gp120 are somewhat similar, manifesting as either the common rotation of the inner and outer domains or expansion/contraction of the CD4-binding cavity. However, the observed differences in fluctuation direction of the peripheral loops, in particular the V1/V2 region between these two forms gp120, possibly have distinct consequences for gp120 conformation. Visual check of the representative structures extracted from free energy basins/minima of FELs reveals that the conformational transition from the liganded to the unliganded states did indeed occur for the CD4-free gp120, but not for the CD4-complexed gp120 during simulations; moreover, the major conformational differences are reflected in the relative orientation of V1/V2 with respect to the gp120 core. As a result, we conclude that the V1/V2 region is the major structural determinant for gp120 transition between different states/substates and that CD4 binding greatly weakens the reorientation ability of V1/V2, and, as such, prevents the transition to the unliganded state. In the absence of CD4, the spontaneous conformational transition of gp120 from the liganded state to the unliganded state supports the proposed conformational selection mechanism for CD4 binding. Additionally, the enhanced mobility of the bridging sheet and certain β-strands of the outer domain upon CD4 removal likely destabilizes certain antibody neutralization epitopes, thus providing a possible explanation for the avoidance of antibody-mediated neutralization of gp120 in the liganded state without CD4 occupation. We suggest that the discovery and development of small molecules capable of restraining V1/V2 reorientation and locking gp120 conformation in either the liganded or the unliganded states may be a promising strategy to control HIV-1 infection.
